# The Pandora’s Box of Hypertensive Heart Disease in Women

**DOI:** 10.1016/j.jacadv.2024.101255

**Published:** 2024-09-06

**Authors:** Sharon L. Mulvagh, Robert L. Stewart, Evan G. Losier

**Affiliations:** a Division of Cardiology, Dalhousie University, Halifax, Nova Scotia, Canada; b Department of Cardiovascular Medicine, Mayo Clinic, Rochester, Minnesota, USA

**Keywords:** echocardiography, hypertension, women

**Figure:**
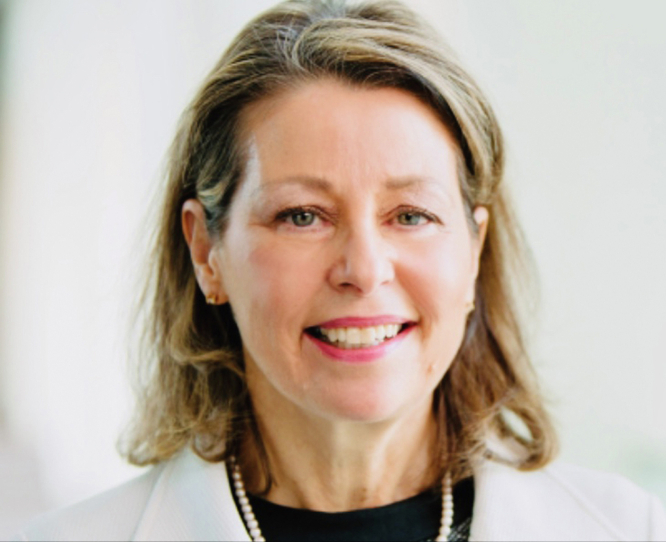

Recently, there has been significant advancement in the recognition of sex and gender-based differences in cardiovascular health and disease. However, while hypertension remains the most common medical diagnosis and modifiable cardiovascular risk factor regardless of sex, a sex- and gender-based approach to diagnosis and management of hypertension has not been explored.^1^, ^2^, ^3^, ^4^ Of note, although the terms sex and gender are used interchangeably in the literature, it should be recognized that sex is a biologic construct, while gender is a social construct; analysis and reporting frequently blur these definitions, as exemplified in this editorial, due to imprecision of source documents.

Autonomic and hormonal function are key contributors to the regulation of blood pressure (BP).^3^^,^^4^ Females experience unique events over the course of their lifespan related to reproductive biology including menarche and menstruation, pregnancy, and menopause. After the onset of menopause, hypertension is more prevalent in females than males.^3^^,^^4^ Despite this, underdiagnosis of hypertension is more common in women than men, and when women are treated with antihypertensive medications, it has been found to be less well controlled than men.^3^^,^^4^ Additionally, women have a unique profile when it comes to efficacy and adverse effects of some antihypertensive medical therapies.^4^ There is also a sex-specific cardiac response to chronic hypertension and pressure overload.^4^ It was initially demonstrated within the analysis of the Framingham Heart Study that women with systolic hypertension experience higher rates of hypertensive left ventricular concentric remodeling and hypertrophy,^5^ a finding has since been corroborated in several observational studies.^6^, ^7^, ^8^ Once established, hypertensive left ventricular hypertrophy (LVH) is less modifiable by antihypertensive treatment in females than males.^8^ Although the overall risk for cardiovascular disease is lower in premenopausal women, this protection is lost after the onset of menopause. Interestingly, it has been shown that the presence of LVH in hypertension offsets the female sex-protection in cardiovascular risk, such that among hypertensive subjects with LVH, both sexes have comparable cardiovascular risk.^9^ It has also been observed that although healthy young women have lower BP than men at similar age, they experience a steeper increase in BP from the third decade of life onward, and women with hypertension more often develop heart failure with preserved ejection fraction and atrial fibrillation, whereas men more often develop acute myocardial infarction and heart failure with reduced ejection fraction.^4^^,^^9^^,^^10^ While sex-related differences in hypertension and left ventricular geometry have been clearly demonstrated,^5^, ^6^, ^7^, ^8^ to date these observations have not translated into mechanistic understandings for these differences, potentially leading to the creation of sex-specific diagnosis or management guidelines in clinical practice. This is primarily due to a lack of sex- and gender-specific analysis and reporting in large clinical trials on treatment and outcomes in hypertension.

In this issue of *JACC: Advances,* Canciello et al^11^ add to the substantial data supporting sex-based differences in hypertensive heart disease by demonstrating the longitudinal impact of hypertension on ventricular morphology and remodeling. This study reports on a post-hoc sex-disaggregated analysis of echocardiograms from 6,427 patients (43% female) in the Campania Salute Network observational registry of hypertensive patients and found over twice the rate of left ventricular remodeling and hypertrophy in females at both baseline and follow-up (mean of 6.1 years) when compared to males. These increased rates of abnormal geometry were found despite exclusion of patients with established cardiovascular disease or significant chronic kidney disease. Female sex was independently associated with abnormal left ventricular geometry in multivariate analysis with an odds ratio of 2.36 (95% CI: 2.12-2.62; *P* < 0.001).

An unexpected result was that the majority of females with abnormal geometry had eccentric hypertrophy, a finding previously thought to be more common in males.^12^ No clear hypothesis was given for this observation, but it is possible that this may represent a longer duration of undertreated hypertension, and an associated increased predisposition to subclinical microvascular coronary artery disease in this female population, leading to more extensive fibrotic changes and eccentric dilation. In a recent study evaluating altered left ventricular geometry using cardiac magnetic resonance imaging and adjusted for epicardial coronary artery disease, it was shown that concentric hypertrophy was associated with increased all-cause mortality in both sexes, while eccentric hypertrophy was associated with increased all-cause mortality only in females.^12^ Bairey Merz et al^13^ pointed to the disproportionate burden of microvascular coronary dysfunction in females as a possible explanation for the increased adverse mortality.

Although observational studies of this nature cannot determine causal relationships, Canciello et al^11^ report findings that are in keeping with previous similar cross-sectional studies and uniquely add to this growing body of information by providing longitudinal evidence for persistent sex disparities over time, shedding light on the prognostic importance of early identification of these sex-specific morphologic changes in response to altered physiology using the readily accessible tool of echocardiography. In addition to LVH, which has been considered the cardinal echocardiographic biomarker of pressure overload and associated cardiac damage, strain assessment of the left ventricle and left atrium are emerging areas of exploration providing more sensitive markers of hypertensive changes preceding chamber enlargement or hypertrophy and may eventually play a greater role in earlier recognition of cardiac alterations and response to antihypertensive treatment.^14^ Sex-differences have been identified in both ventricular and atrial strain, and the establishment of sex-specific strain reference values would be an essential step for unlocking strain assessments as potential tools aiding in mechanistic understanding and management.^15^^,^^16^

Limitations to retrospective analysis of observational data are acknowledged by the group, including the undefined role of potential underlying subclinical coronary artery disease and lack of detail regarding individual antihypertensive therapies. In addition, there is no data addressing the potential for associations with cardio-obstetric sex-specific cardiovascular risk factors such as hypertensive pregnancy disorders (preeclampsia, eclampsia), polycystic ovary syndrome, menopausal status, and hormonal drug therapies.

In summary, this latest observational study by Canciello et al^11^ confirms sex disparities in the physiologic response to hypertension adding to the growing body of research demonstrating sex-specific differences of hypertensive heart disease and prognostic implications of these differences, and in so doing raises many questions that urgently require answers. Why do female hypertensive patients exhibit a higher propensity for left ventricular remodeling than their male counterparts? Is eccentric hypertrophy truly more prevalent in females? If so, what is the adaptive impact and association with increased clinical prevalence of coronary microvascular dysfunction and heart failure with preserved ejection fraction in females? What new pharmacologic strategies could be indicated? There is a clear need to better understand the underlying mechanisms of these sex differences in adaptive ventricular geometry in order to develop optimal sex-specific diagnostic and prognostic strategies to guide the optimal management of hypertension in women. Moreover, this work underscores the importance of considering sex- and gender-specific factors in risk assessment and management strategies and demonstrates that analysis of data through a sex-specific lens is essential to our understanding of cardiovascular pathophysiology. Integrating sex-specific risk factors into risk stratification models is a crucial step toward more tailored and effective clinical interventions.

## Funding Support and Author Disclosures

Dr Mulvagh is a consultant for Novo Nordisk (no relevance). All other authors have reported that they have no relationships relevant to the contents of this paper to disclose.
